# Correction: Swimmer’s itch control: Timely waterfowl brood relocation significantly reduces an avian schistosome population and human cases on recreational lakes

**DOI:** 10.1371/journal.pone.0304376

**Published:** 2024-05-21

**Authors:** The PLOS ONE Staff

## Notice of Republication

This article was republished on May 6, 2024, to correct an error in the PDF version. A portion of the results section mistakenly appeared as a footnote in Table 4 in the PDF. The publisher apologizes for this error. Both the originally published, uncorrected article and the republished, corrected version are provided here for reference.

## Supporting information

S1 File Originally published, uncorrected article. (PDF)

S2 File Republished, corrected article. (PDF)
