# Supplementary figures and images for: Swimmer’s itch control: Timely waterfowl brood relocation significantly reduces an avian schistosome population and human cases on recreational lakes
Source: PLoS One. 2024 Feb 15;19(2):e0288948. doi: 10.1371/journal.pone.0288948 (PMC10868848; doi:10.1371/journal.pone.0288948)

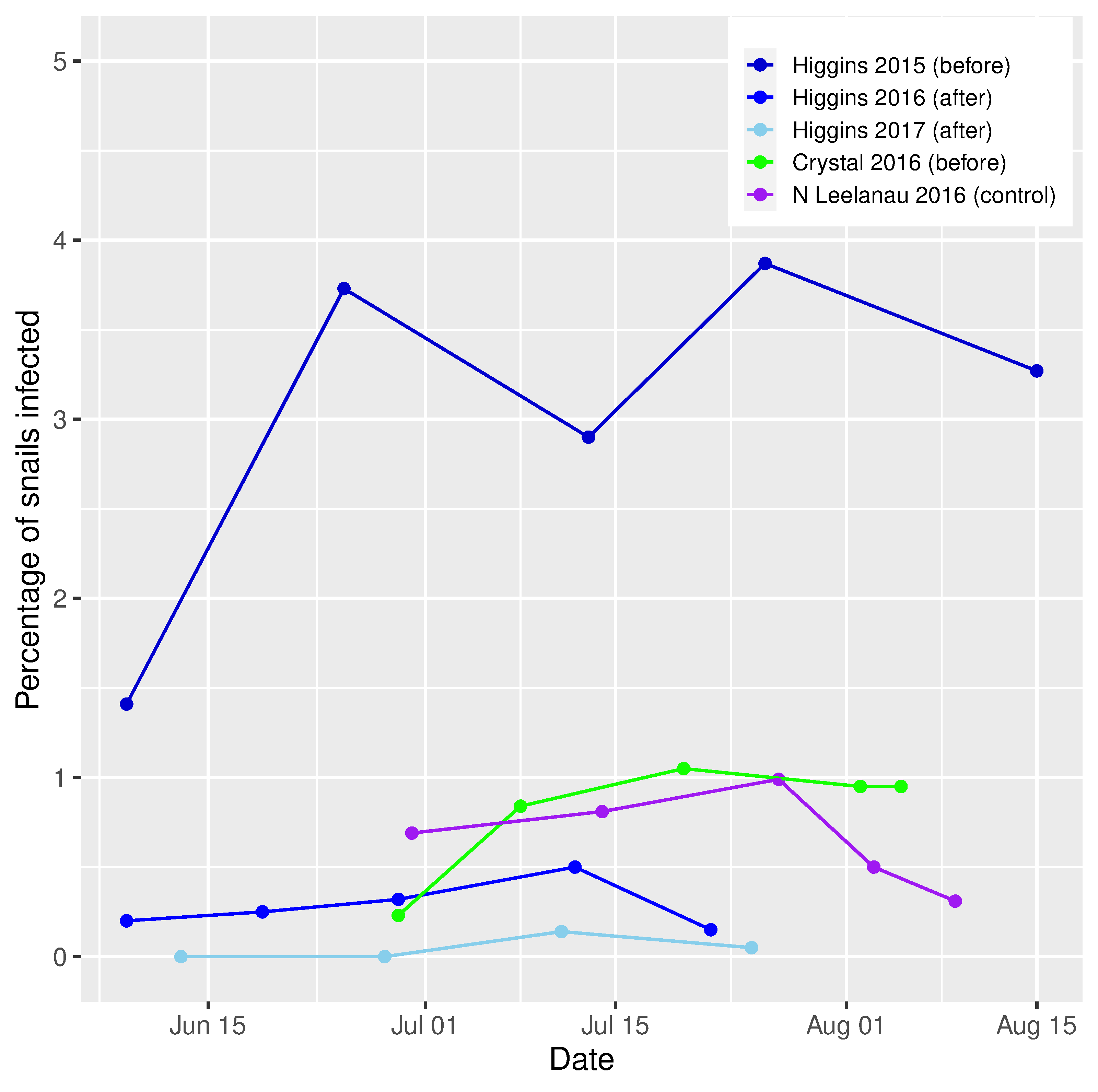

Supplement: S1 Fig — Higgins 2016 and 2017 collections (in lighter shades of blue) are after mitigation had begun and highest levels were still detected in mid-July. Consequently, all later collections (2018–2020) occurred in mid-July. Data represent 44310 snails examined. (TIF) [file pone.0288948.s001.tif]
